# Beyond coronary stenosis: Can high-risk plaque features redefine risk stratification in low to intermediate risk patients?

**DOI:** 10.1007/s00330-026-12343-8

**Published:** 2026-03-05

**Authors:** Costanza Lisi, Federica Catapano

**Affiliations:** 1 Department of Biomedical Sciences, Humanitas University, Milan, Italy; 2 IRCCS Humanitas Research Hospital, Milan, Italy

In recent years, coronary computed tomography angiography (CCTA) has progressively evolved from a purely anatomical test aimed at detecting luminal stenosis into a comprehensive tool for coronary artery disease (CAD) characterization and risk stratification [1]. This paradigm shift reflects a growing recognition that the burden and biological behavior of atherosclerosis extend well beyond the degree of luminal narrowing [2]. Within this context, the identification of high-risk plaque (HRP) features on CCTA has emerged as a promising strategy to refine prognostication and potentially guide preventive therapies [3]. Despite robust pathophysiological rationale and consistent associations reported in observational studies, the clinical role of HRP features remains debated. Key unresolved questions include whether HRP confers independent prognostic value beyond traditional cardiovascular risk factors and obstructive CAD, and whether its identification meaningfully improves patient management in contemporary practice.

The prespecified secondary analysis of the DISCHARGE trial by Szilveszter et al [4] directly addresses these issues in a large, multicenter, pan-European cohort, providing timely and clinically relevant insights. The analysis included 1745 patients with stable chest pain and low-to-intermediate pretest probability of CAD undergoing CCTA. The authors demonstrate that HRP features—defined as the presence of positive remodeling, low-attenuation plaque, the napkin-ring sign, or a coronary artery calcium score ≥ 400 Agatston units—are independently associated with both major adverse cardiovascular events (MACE) and an expanded composite endpoint. Importantly, this association persists after adjustment for conventional cardiovascular risk factors. Notably, patients with HRP in the absence of obstructive CAD exhibited a risk of adverse events comparable to those with obstructive CAD alone, while the coexistence of both conferred the highest risk.

These findings provide several important contributions to the current literature. First, they extend prior observations from studies such as PROMISE [5] and SCOT-HEART [6] by confirming the prognostic relevance of HRP features within the rigorous framework of a randomized, pragmatic clinical trial. Second, the inclusion of patients initially referred for invasive coronary angiography but managed with a CT-first strategy reflects a real-world population in whom risk stratification is particularly consequential. Moreover, the study highlights the incremental value of plaque characterization over stenosis-based assessment, with the addition of HRP features improving model discrimination for adverse events.

Beyond statistical associations, the clinical implications of these results warrant careful consideration. The observation that HRP alone carries a risk comparable to obstructive CAD challenges current decision-making pathways that largely prioritize luminal narrowing. From a conceptual standpoint, this supports a shift toward a more biology-driven interpretation of coronary atherosclerosis, where plaque vulnerability and total plaque burden play a central role. From a practical perspective, it raises the question of whether patients with non-obstructive but high-risk plaques should be managed more aggressively with preventive therapies, closer surveillance, or both.

At the same time, several aspects temper immediate clinical translation. Event rates in this cohort were relatively low, reflecting contemporary preventive strategies and the low-to-intermediate risk profile of the population. Moreover, HRP assessment relied on site-level qualitative interpretation rather than quantitative plaque analysis, potentially limiting reproducibility across centers [7]. The inclusion of a high coronary calcium score as part of the HRP definition, while pragmatically justified, also underscores the challenge of differentiating plaque vulnerability from overall atherosclerotic burden. Although coronary calcium burden emerges as the strongest prognostic predictor in the analysis, the absence of a direct and comprehensive quantification of total atherosclerotic plaque burden on CCTA may represent a significant limitation. In this context, it remains uncertain whether CT-derived HRP features offer incremental prognostic information beyond advanced coronary atherosclerotic disease burden, or whether they primarily reflect the presence of more extensive coronary artery disease. These considerations highlight that, while HRP features clearly refine risk stratification, their role as standalone decision-making tools remains to be fully defined.

Importantly, the DISCHARGE protocol incorporated guideline-oriented preventive recommendations triggered by CT findings, including HRP. This integrated approach likely contributed to the attenuation of absolute event rates and may partially explain differences in hazard ratios compared with earlier studies. Notably, plaque burden is already known to correlate with laboratory biomarkers [8], and long-term evidence from CCTA-guided management strategies has demonstrated a sustained reduction in coronary heart disease death or non-fatal myocardial infarction, despite no increase in invasive coronary angiography or revascularization rates and with persistently higher use of preventive therapies over time [9]. Rather than reducing the value of HRP assessment, this observation reinforces a key message: imaging biomarkers derive their true clinical impact when embedded within structured management pathways. In this regard, the study moves the field beyond prognostic association toward a more translational perspective.

Several future perspectives emerge from this study: standardization of HRP definitions, acquisition protocols, and reporting remains essential to ensure reproducibility and facilitate broader adoption. Quantitative plaque analysis, potentially augmented by artificial intelligence, may improve risk stratification by capturing plaque burden and composition with greater precision. Furthermore, prospective studies are needed to determine whether HRP-guided management strategies translate into improved clinical outcomes and to define actionable thresholds that can be readily applied in daily practice.

In conclusion, this prespecified analysis of the DISCHARGE trial provides compelling evidence that HRP features on CCTA offer prognostic information beyond stenosis and traditional risk factors in patients with stable chest pain. By demonstrating that HRP alone confers a risk comparable to obstructive CAD, the study challenges conventional paradigms and reinforces the value of comprehensive plaque assessment. While further work is required to optimize standardization and clinical integration, these findings represent an important step toward a more nuanced, biologically informed use of CCTA in cardiovascular risk assessment.
